# Primary Progressive Aphasia: Toward a Pathophysiological Synthesis

**DOI:** 10.1007/s11910-021-01097-z

**Published:** 2021-02-04

**Authors:** Justina Ruksenaite, Anna Volkmer, Jessica Jiang, Jeremy CS Johnson, Charles R Marshall, Jason D Warren, Chris JD Hardy

**Affiliations:** 1 Dementia Research Centre, Department of Neurodegenerative Disease, UCL Queen Square Institute of Neurology, University College London, 8 – 11 Queen Square, London, WC1N 3BG UK; 2 Division of Psychology and Language Sciences, University College London, London, UK; 3 Preventive Neurology Unit, Wolfson Institute of Preventive Medicine, Queen Mary University of London, London, UK

**Keywords:** Primary progressive aphasia, Frontotemporal dementia, Alzheimer’s disease, Logopenic aphasia, Semantic dementia, Progressive nonfluent aphasia, Physiology

## Abstract

**Purpose of Review:**

The term primary progressive aphasia (PPA) refers to a diverse group of dementias that present with prominent and early problems with speech and language. They present considerable challenges to clinicians and researchers.

**Recent Findings:**

Here, we review critical issues around diagnosis of the three major PPA variants (semantic variant PPA, nonfluent/agrammatic variant PPA, logopenic variant PPA), as well as considering ‘fragmentary’ syndromes. We next consider issues around assessing disease stage, before discussing physiological phenotyping of proteinopathies across the PPA spectrum. We also review evidence for core central auditory impairments in PPA, outline critical challenges associated with treatment, discuss pathophysiological features of each major PPA variant, and conclude with thoughts on key challenges that remain to be addressed.

**Summary:**

New findings elucidating the pathophysiology of PPA represent a major step forward in our understanding of these diseases, with implications for diagnosis, care, management, and therapies.

## Introduction: the Scope of the Problem

The language-led dementias or ‘primary progressive aphasias’ (PPA) are a unique group of neurodegenerative proteinopathies that share a propensity to target language networks of the human brain, with symptom onset often occurring before the age of 65. Although clinical disorders that would now be termed PPA have been accurately described since the nineteenth century, only in the last few decades have the complexity and variability of these diseases become widely appreciated [1•]. Arguably more than any other condition, PPA has transformed our picture of neurodegenerative diseases as disorders of selective neural vulnerability and targeted network disintegration [2, 3]. However, despite considerable progress in detailing the clinical features, structural brain anatomy, and histopathology of PPA, many challenges remain. The current (2011) consensus criteria for syndromic diagnosis based on clinical, neuropsychological, and neuroimaging features [4] do not capture a significant proportion of the clinical spectrum [5]. We lack a clinical staging system capturing verbal and non-verbal symptoms across the main PPA variants; or robust, dynamic biomarkers of disease progression and prognosis. The associations between pathogenic protein deposition and clinical phenotypes are poorly defined; and above all, effective treatments for preserving day-to-day functioning and modifying disease course have not yet been developed. These issues are crucial not only for people living with PPA but as a paradigm for similar challenges spanning the range of neurodegenerative disease.

Here, we argue that these problems might be addressed by an improved pathophysiological understanding of PPA. In support of our argument, we consider recent developments in the field to show how a pathophysiological paradigm might inform clinical and molecular characterisation, prognosis, novel biomarker development, and therapies in PPA.

## The Challenge of Clinical Diagnosis: Detection and Definition

**Fig. 1:**
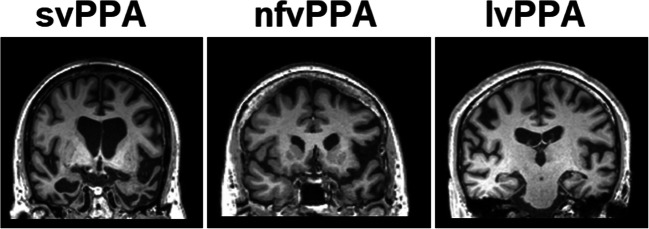
Neuroanatomical profiles of the major syndromes of primary progressive aphasia. Panels present T1-weighted coronal brain MRI sections of patients with typical syndromes of semantic variant primary progressive aphasia (svPPA), nonfluent/agrammatic primary progressive aphasia (nfvPPA), and logopenic variant primary progressive aphasia (lvPPA). Brain images are presented with the left hemisphere on the right. The svPPA scan shows asymmetric (predominantly left-sided) anterior inferior and mesial temporal lobe atrophy; the nfvPPA scan shows asymmetric (predominantly left-sided) inferior frontal, insular, and anterior-superior temporal gyrus atrophy, while the lvPPA scan shows asymmetric (predominantly left-sided) temporo-parietal junction atrophy

Early and accurate diagnosis of PPA is crucial to allow patients and families to plan for the future and to access the support and services they need. Moreover, it is the gateway to effective management, which is likely to include disease modifying therapies in the foreseeable future. Currently, three main clinico-anatomical syndromes of PPA are recognised in consensus diagnostic criteria [1•, 4]. First, nonfluent/agrammatic variant PPA (nfvPPA), characterised by insidious impairment of speech sound and connected speech production and subsequently other language output channels, associated with dysfunction and atrophy predominantly involving left peri-Sylvian cortices centred on inferior frontal gyrus and anterior insula [1•]. Second, semantic variant PPA (svPPA), characterised by erosion of knowledge about words, and ultimately objects and concepts across all sensory modalities, associated with dysfunction and atrophy of the semantic appraisal network, most severe in antero-mesial temporal lobe and generally initially predominantly left-sided [1•, 6]. Third, logopenic variant PPA (lvPPA), characterised by progressive anomia and phonological processing, in particular auditory verbal working memory, associated with dysfunction and atrophy usually predominantly involving left temporo-parietal cortices [7]. Representative coronal sections of T1-weighted MRI scans for each major PPA subtype are shown in Fig. 1.

Applying these diagnostic criteria represents a significant challenge, even for experts. We have previously produced a ‘diagnostic roadmap’ to help the decision-making process [1•]. However, whilst assessment of some domains (e.g. naming, repetition) can be achieved with relative ease, other components such as assessing apraxia of speech, object knowledge, and ‘frank’ agrammatism are harder to operationalise [1•]. Whilst semantic variant PPA is widely recognised as a coherent diagnostic category [8], fragmentary syndromes fall under the umbrellas of nfvPPA and lvPPA. Within nfvPPA, there may be a distinct group of people with ‘pure’ apraxia of speech [9] (which can itself be fractionated into phonetic, prosodic, and mixed subvariants [10]), and cases of pure agrammatism have also been identified [11•]. Primary progressive dynamic aphasia has also been reported as a subvariant of nfvPPA [12, 13], and several cases of nfvPPA with prominent auditory processing symptoms have also been identified (see section below). In logopenic variant PPA, research has identified three different subvariants based on core deficits of (i) single-word comprehension, (ii) repetition, and (iii) confrontation naming, each associated with specific atrophy profiles [14•]. Moreover, vocabulary loss (a core feature of svPPA) may be relatively common in nfvPPA [15]. A lack of understanding of the underlying pathophysiology in PPA syndromes is a common theme that makes principled diagnostic classification difficult. These difficulties with clinical differentiation of PPA variants have led to the suggestion that common syndromes across the frontotemporal dementia (FTD) spectrum (including nfvPPA and svPPA) are not discrete in their clinical features, existing in a multidimensional space [8•]: according to this formulation, diagnostic criteria for PPA may need to be broadened to include intermediate diagnoses [16].

Whilst typically being regarded as young-onset dementias, recent epidemiological evidence suggests that there is a cluster of older people in their eighth or ninth decade who develop nfvPPA [17••]: this group is likely to be significantly under-diagnosed. There can also be significant problems with symptom onset: whilst the major PPA variants can initially present as selective deficits in specific domains, over time, a ‘mixed’ phenotype is likely to emerge. This mixed phenotype may, however, be present from the initial stages of the disease [18], creating considerable diagnostic challenges. The opposite problem is true too: leading symptoms in all PPA variants can be extremely subtle and are not always well captured by the current criteria, which focus on established disease [4]. Moreover, despite being characterised as ‘language-led’ disorders, patients and carers often report extra-linguistic features, for which supporting research evidence is now emerging, including auditory dysfunction (see section below) and abnormal nonverbal behaviours [19, 20]. Given that early detection and diagnosis is likely to be vital for successful therapies, a reformulation of the criteria around identification of the very earliest symptoms would be timely.

## The Challenge of Clinical Prognosis: Assessing Disease Stage and Activity

Prognosis and anticipation of deficits later on are hugely important for patients and carers, yet robust and reliable ways of assessing disease stage and rate of progression are currently lacking. Rates of clinical evolution differ between the major PPA variants and may be more rapid in nfvPPA [21]. A significant proportion of people with nfvPPA will develop Parkinsonism, which seems more likely to develop in those people who have prominent apraxia of speech [22, 23••], but clear early pathophysiological markers of anticipated trajectories across the PPA spectrum are needed. A related issue is the lack of widely applicable severity measures across PPA syndromes: ‘standard’ tools are heavily focused on symptoms associated with typical Alzheimer’s disease and may significantly over-emphasise severity in PPA due to heavy language components, as with the Mini-Mental State Examination, whilst the Frontotemporal Dementia Rating Scale [24] was not developed for people with lvPPA, and the Progressive Aphasia Severity Scale [25] does not take account of non-language symptoms. The recently developed Mini Linguistic State Examination [26•] may help to address some of these issues, but there remains a clear need for a symptom-led staging system for each of the major PPA variants that can capture the range of language and non-verbal symptoms that are present in each subtype [1•].

Evidence suggests a clear rationale for a personalised approach: language functions are highly dependent on life trajectory, and intriguing links have been identified between PPA and dyslexia [27]. Very recent work suggests that bilingualism may delay the onset of lvPPA [28]. Much needs to be done in identifying the earliest symptoms of PPA: in svPPA, for example, the patient will virtually always have established focal atrophy on MRI at the time of diagnosis, and standard psychometric tests are often subject to floor/ceiling effects: svPPA patients typically struggle to name more than a single item correctly on the graded naming test [29••]. Identification and stratification into clinical trials at the very earliest stage of disease will be vital if treatments are to be effective. The use of ecologically relevant measures that quantify aspects of speech output have garnered recent interest in terms of both diagnosis and disease tracking from early stages [30–33], leading to the suggestion that automated analysis of speech might be used as a ‘verbal thermometer’ for PPA and FTD [34]. There is also considerable interest in the use of ‘wet biomarkers’ in measuring disease burden and intensity. For instance, neurofilament light chain protein has emerged as a measure of disease intensity across the FTD spectrum, regardless of the underlying pathology [35]. However, despite these strides forward in tracking biomarkers of disease intensity, information is currently lacking about symptom management, quality of life, and palliative care in late-stage PPA.

## The Challenge of Molecular Diagnosis: Physiological Phenotyping of Proteinopathies

One of the most ambitious, but most important, challenges in neurodegenerative disease research is to identify how pathogenic proteins give rise to complex phenotypes. The major molecular histopathological associations differ between the major syndromes of PPA [22, 23••, 36, 37•]: nfvPPA is most often associated with primary tauopathies, svPPA is closely associated with TDP-43 (type C) pathology, and lvPPA with Alzheimer pathology, leading to the proposal that the PPA may constitute ‘molecular nexopathies’ [2], i.e. specific conjunctions of macroscopic network characteristics and pathogenic protein properties. This formulation has received empirical support [22, 38, 39]. However, there is not complete concordance between phenotype and pathology: svPPA can more rarely be caused by tau or Alzheimer pathologies [22, 37•, 40]; nfvPPA is sometimes caused by Alzheimer’s disease, or TDP-43 (type A or B) [22, 37•, 41, 42]; lvPPA has been associated with dementia with Lewy bodies[43] and TDP-43 (type A) [41]; and an lvPPA-like phenotype has been consistently identified in people with mutations in the progranulin gene [44, 45]. Emerging evidence suggests that specific language network vulnerabilities caused by genetic, developmental, and/or lifestyle factors may determine why some people develop a PPA phenotype in the context of a specific proteinopathy [27, 46, 47]. Age at onset may also influence phenotypic expression [48]. Clinical and neuropsychological tests may reveal certain clues as to the underlying proteinopathy in PPA: non-verbal episodic memory deficits are associated with Alzheimer pathology [49], whilst Parkinsonism (often evolving into a progressive supranuclear palsy/corticobasal syndrome) and apraxia of speech are typically associated with a tauopathy [22, 23••]. Motor neuron disease features accompanying nfvPPA and svPPA may be under-recognised and predict underlying histopathology [50, 51].

Whilst most PPA syndromes are sporadic, making presymptomatic diagnosis difficult, rarer cases of progranulin-associated aphasia [44, 45] and other genetic forms of PPA could represent important ‘test’ cases. Communication functions are uniquely complex, and carefully designed tests that can tax the integrity of these vulnerable networks might be used as ‘stress tests’ of early disease. In PPA, paradigms of artificially degraded speech may hold promise in this regard [52••], as tests of degraded visual object processing have emerged as early markers of cognitive decline in the context of Lewy body disease [53]. Other novel tests probing altered socio-emotional reactivity [54, 55], autonomic functions [56, 57••], sleep symptoms [58], and other functions may also track protein-linked dysfunction across a wider range of disease stages than conventional psychology tests.

A current major focus is in the use of in vivo positron emission tomography imaging. However, whilst there have been promising developments with radioligands showing increased binding to pathologically affected regions in tauopathies, the specificity of these ligands is typically unsatisfactory [59]. Developments are ongoing to develop ligands that may bind with greater specificity to tau and TDP-43, whilst markers of neuroinflammation may represent promising tools for tracking disease severity regardless of proteinopathy [60, 61]. Novel, connectivity-based analysis approaches may help to elucidate mechanisms of pathogenic protein spread: recent work using spectral dynamic causal modelling has suggested that attenuation of inhibitory connectivity in antero-mesial temporal lobes may help drive TDP-43 (type C) pathogenic protein spread in svPPA[62••]. Innovative magnetoencephalography [63–66] and functional magnetic resonance imaging [29••, 54] paradigms have also shown utility in delineating unique neurophysiological signatures of functional connectivity and plasticity in major PPA variants: these techniques may hold promise as very early markers of neurodegeneration, when atrophy is not prominent [67].

## Evidence of Central Auditory Impairment on Psychoacoustic Tests

Language and auditory functions are closely interrelated [68], meaning that auditory measures may represent useful probes of integrity of the relevant networks in PPA [69]. Indeed, language output deficits in all three major PPA syndromes are likely to be significantly influenced by disordered complex sound processing and understanding: an emerging picture in PPA is of fundamental deficits in central auditory perception [70, 71]. People with svPPA show profound environmental sound agnosia [71] and phonagnosia [72], alongside symptoms of tinnitus and hyperacusis [73]. Deficits at a relatively ‘early’ level of auditory processing have been identified in nfvPPA, including sound detection [74••]; reduced activity in primary auditory cortex[29••]; pitch and timbral pattern processing [71]; and processing of rhythmicity of tone sequences [29••, 75••, 76]. Accurate speech perception relies on successful integration of bottom-up sensory information with top-down predictive processing, which is also thought to be impaired in nfvPPA [77•]. Logopenic variant PPA is associated with deficient processing of phonemes [29••, 78••]. One parsimonious explanation for the phonemic errors made in speech output in lvPPA is that these relate to a general impairment in phonemic representation, though the association between phonemic input and output errors is yet to be explored experimentally. Patients with lvPPA also show pronounced difficulties understanding degraded speech [52••], which may reflect a more general deficit in terms of parsing the auditory environment (‘auditory scene analysis’)—this has not been tested experimentally but accords with findings in typical Alzheimer’s disease and posterior cortical atrophy (a visuospatial form of Alzheimer’s disease) suggesting that damage to posteromedial cortices may underpin these deficits across the AD spectrum [79, 80].

Atypical presentations in the PPA/FTD spectrum manifesting as very early problems with auditory processing have also been identified, including progressive pure phonagnosia [46, 81], progressive word deafness [82–84], and generalised auditory agnosia [85]. Taken together, these recent findings suggest that the PPA might be characterised as pervasive ‘communication’ disorders that go beyond language. Tests of auditory processing could have considerable advantages over tests measuring language functions: they are relatively easy to measure and administer, and less linguistically/culture-bound than language tests, making them attractive as outcome measurements in future clinical trials.

## The Challenge of Treatment: Optimising Function And Changing Disease Course

Unfortunately, no disease-modifying treatments for any PPA variant currently exist, though clinical trials are now underway for tauopathies (for a review, see[86]). Disease modification approaches will necessarily be protein-directed, rather than PPA specific, re-emphasising the need for some kind of molecular biomarker. Physiological understanding may improve prediction of molecular pathology, but it is also important for monitoring response to treatment and demonstrating efficacy in these syndromes specifically. Without disease-modifying therapies, non-pharmacological approaches to PPA management are of considerable importance, and non-invasive brain stimulation techniques to ameliorate specific symptoms across PPA variants have received much recent attention. Transcranial direct current stimulation as an adjunct to traditional speech and language therapy has shown some promise in PPA [87, 88], and there have been some reports of success with transcranial magnetic stimulation in case studies and small cohorts [89–91]; clinical trials are currently underway.

People with PPA may benefit from physiologically informed cognitive rehabilitation strategies, akin to strategies designed to enhance neuroplasticity after stroke aphasia [92, 93]: recent work suggests that people with all major forms of PPA have retained capacity for perceptual learning of degraded speech [52••], and that patients with nfvPPA show preserved faculty for artificial grammar learning [94], suggesting the need for future trials focused on exposure-based approaches to rehabilitation of agrammatism and degraded speech perception. In svPPA, right-lateralised brain regions show elevated activity in magnetoencephalography when listening to spoken words, whilst dorsal regions appear to compensate for damaged ventral regions when patients read irregular words, together suggesting a degree of functional plasticity in brain networks with relatively preserved integrity [63, 64].

A ‘one size fits all’ approach is unlikely to be effective in PPA, and prevailing research suggests that pathophysiologically targeted speech and language therapy interventions may yield most success [95, 96]. In svPPA, improvement in vocabulary after naming intervention has been observed alongside activation of larger networks in bilateral brain regions on functional neuroimaging [97–99, 100••, 101], though word-retrieval therapies are only likely to yield benefit on trained items (transfer to untrained items is more likely to be seen in lvPPA [101]). In nfvPPA, script training has been shown to improve automation of speech production, resulting in immediate and long-term outcomes in terms of intelligibility and grammaticality, up to 1 year after treatment [102].

In recent years, there has been an increase in research focusing on compensatory, or functional approaches to symptom management for people with PPA [103]. These approaches focus on compensating for the speech and language difficulties through use of strategies, environmental supports, or augmentative alternative communication aids. This reflects the practice of clinical speech and language therapists who report prioritising communication partner training approaches when with people with PPA and their family members [104–107]. This approach targets everyday conversation between a person with PPA and a family member or carer, and is underpinned by an assessment of strategies that facilitate communication (e.g. gesture) and those that act as barriers (e.g. completing a person’s sentence without an invitation to do so, or abrupt topic changes) [105, 108].

Despite this emerging evidence base, speech and language therapists across the UK report barriers to people with PPA accessing their services including a lack of knowledge amongst referrers about the benefits of speech and language therapy for PPA, restrictive service criteria and commissioning limitations, and a lack of confidence [106, 107]. These issues are reflected internationally, with similar issues reported across the USA and Australia [105], highlighting a need for further work in developing the relevant research evidence to underpin care pathways to support referrers, with a complementary focus on education of health care professionals (including speech and language therapists) and commissioners. Dedicated PPA support groups have an important role to play in comprehensive PPA care [109]. In the UK, Rare Dementia Support run the national PPA Support Group (www.raredementiasupport.org) and there are many others in different countries.

## An Interim Pathophysiological Synthesis of PPA

**Fig. 2:**
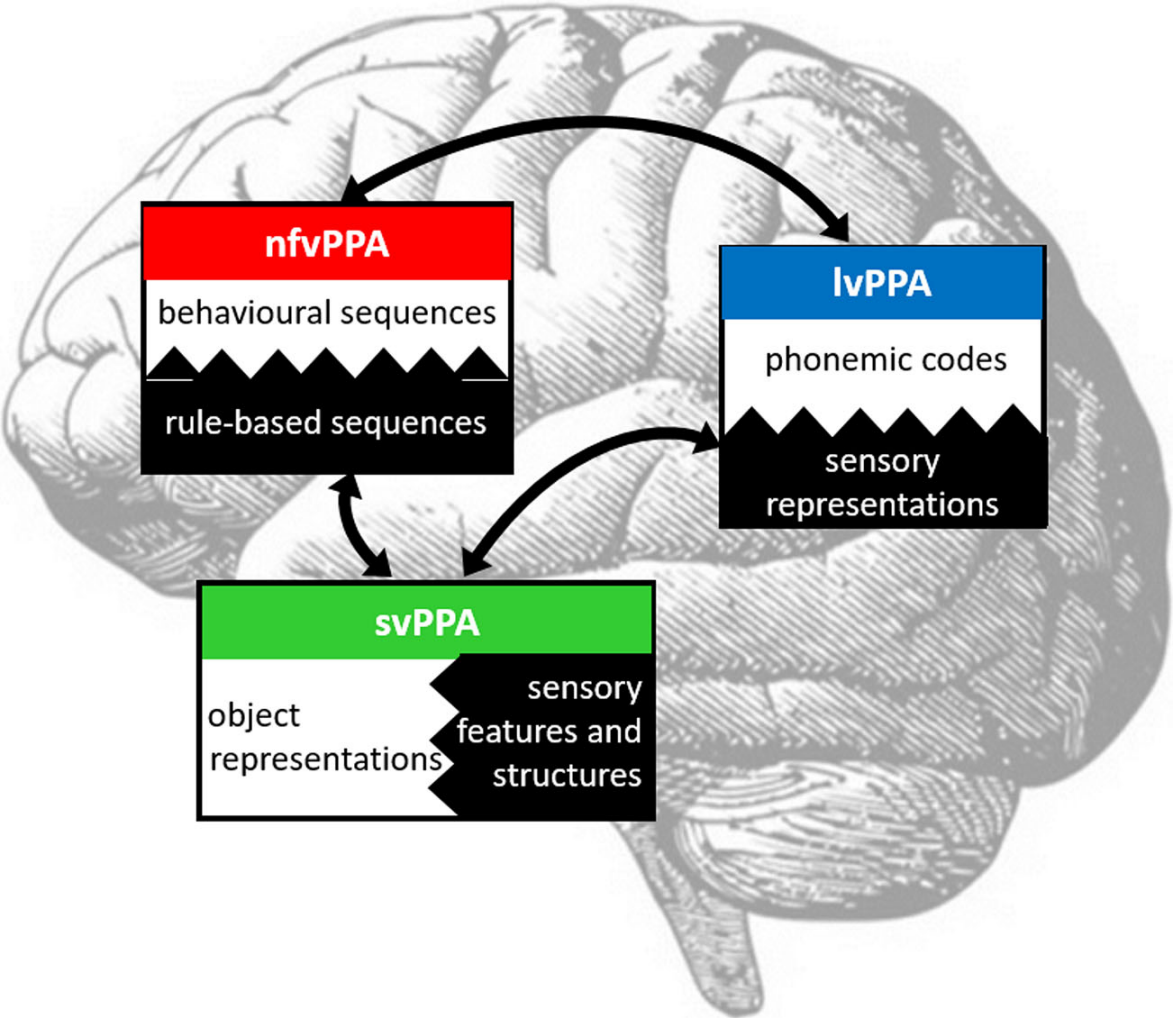
Proposed pathophysiological synthesis of primary progressive aphasias. The figure diagrams core neural processes proposed to be targeted in each of the canonical syndromes of primary progressive aphasia, projected on a lateral view of the left cerebral hemisphere. Oblongs signify core neural processing modules or circuits within the language network: each instantiates a key template-matching operation in which incoming data (represented by black hatching) is iteratively reconciled with prior predictions and transformed into an output (predictive decoding; see text). The bidirectional arrows represent the reciprocal exchange of data and predictions between core processing modules. Processing modules are organised hierarchically, in that incoming sensory representations arriving at temporoparietal junctional cortex (blue) are transformed into increasingly abstract conceptual representations in anterior temporal cortex (green) and may ultimately be used in generating a motor output via anterior peri-Sylvian mechanisms (red). However, extensive dynamic interactions between processing modules are essential to the normal operation of the language system. Note that the neuroanatomical loci of the processing modules designated here correspond only loosely to ‘Broca’s area’, ‘Wernicke’s area’, and other structures emphasised in classical (vascular) aphasiology; the primary progressive aphasia are essentially network-based disorders. lvPPA, logopenic variant of primary progressive aphasia; nfvPPA, nonfluent-agrammatic variant of primary progressive aphasia; svPPA, semantic variant of primary progressive aphasia

We argue that the rich phenomenology of PPA is underpinned by core pathophysiological processes that differ between canonical PPA syndromes (diagrammed schematically in Fig. 2).

The motivation for moving beyond traditional neurolinguistics accounts of these syndromes to address core pathophysiological mechanisms is fourfold. Firstly, a pathophysiological perspective may explain certain associated clinical features of PPA syndromes (such as nonverbal auditory dysfunction and complex nonverbal behavioural changes) that are difficult to reconcile with purely neurolinguistic accounts. Secondly, it grounds PPA in neurobiological mechanisms of language that emphasise neural circuit function (see for example [110]), thereby building bridges to the molecular pathological processes that target these circuits and potentially linking complex disease phenotypes to the activity of pathogenic proteins. Thirdly, it opens up fresh avenues for characterising these diseases using physiologically informed methods, such as animal models, artificial neural networks, and functional neuroimaging. Finally, it promises to motivate the development of new, cross-linguistic, physiological biomarkers for detecting and tracking the effects of culprit pathogenic proteins and therapeutic interventions dynamically and ultimately, to inform the design of novel therapies.

### Features of Language Networks Confer Susceptibility to Neurodegenerative Pathologies

Dynamic transformation of information (from percept to meaning and to action) is integral to the normal operation of the language system. This was implicit in the early models of Lichtheim and others but modern neuroimaging and neurophysiological techniques have elaborated candidate neural mechanisms by which linguistic transformation occurs—in particular, predictive coding and matching of sensory stimuli and motor programmes to stored neural representations or ‘templates’, operating iteratively across hierarchically organised neural circuits [110, 111].

The transformation of speech signals engages highly distributed neural networks and is characterised neurophysiologically by spatio-temporal integration, nonlinear coding, plasticity, and reciprocal interaction between processing stages. These are generic neurophysiological processes that also operate on nonverbal auditory and many other kinds of information [69]. However, the accurate predictive decoding and encoding of verbal messages are so exquisitely dependent on these functional characteristics that any pathology that targets the relevant neural circuitry is likely to have a disproportionate, early impact on speech and language compared with other kinds of signal processing. For example, to fully process a heard signal such as ‘dandelion’ requires parsing of the relevant phonemic objects with high temporal resolution, adaptation to diverse, varying listening conditions (background noise, accent, vocal identity, etc.), and matching to stored associated information that conveys meaning, whilst to deploy it in speech output requires combinatorial sequencing of the constituent phonemes, scheduling, and execution of the corresponding motor programme.

Within this matrix, canonical PPA syndromes affect core neural processes relatively selectively. To the extent that these syndromes tend to be caused by different molecular pathologies, this selectivity is in turn a readout of particular pathogenic proteins and protein configurations that target specific neural circuit elements, in line with the molecular nexopathies paradigm [2, 3] (TDP-43 in semantic appraisal network, svPPA; AD pathology in default mode network, lvPPA; hyperphosphorylated tau in dorsal peri-Sylvian networks, nfvPPA). Following this formulation, the greater clinical and pathological heterogeneity of nfvPPA might reflect the lack of a single coherent ‘nexopathy’ in this syndrome. Certain molecular pathologies may affect more than one core neural process from an early stage, leading to atypical or mixed PPA phenotypes (for example, progranulin mutations may produce widespread degeneration of long-range pathways within the language hemisphere [45]).

### svPPA

This syndrome has been more widely studied from a pathophysiological perspective than other PPA syndromes. A candidate generic mechanism for svPPA is impaired computation of the featural statistics that support categorisation and identification of sensory stimuli, due to degradation of the neural activity patterns corresponding to coherent object and concept templates. This mechanism is suggested by several converging lines of evidence. Neuropsychologically, patients show impaired recognition of idiosyncratic exemplars and inappropriate generalisation between object categories due to over-reliance on superficial sensory (rather than conceptual) similarities, in both verbal and nonverbal domains (e.g. they may misclassify a Manx cat as a dog, or sound the English word sew as ‘soo’ [6, 112]), but also show impaired ability to process higher-order regularities in sensory stimuli such as syllable strings [29••, 76] and may have difficulty distinguishing ‘real’ objects from comparably perceptually complex foils [71]. Neurophysiologically, there is reduced GABA-ergic recurrent inhibition within local circuits of the core semantic appraisal network [62••, 113], predicting a loss of definition of stored neural object templates and erosion of boundaries between object representations. The complex behavioural changes associated with svPPA such as rigidity, disinhibition, and altered dietary preferences might be in part compensatory but could equally reflect altered processing of socio-emotional signals: indeed, behavioural disturbance in svPPA has a neurophysiological substrate in common with impaired object recognition [62••].

### lvPPA

The core mechanism of this syndrome may be impaired activation of phonemic templates, leading to deficient parsing of input sensory signals for transcoding to phonological output. Such a mechanism might account for a number of core features of the syndrome, including anomia, phonological, and neologistic errors in speech and writing and reduced verbal working memory capacity [1•, 7, 114–117]. It could also plausibly underpin recent findings of impaired understanding of degraded speech [52••] and impaired phonemic discrimination [78••] in lvPPA. This mechanism might further account for various bedside observations that are less well characterised. For example, on a phrase repetition task, patients tend to demonstrate not merely a reduced verbal working memory span, but intrusions from previously administered phrases and ‘hunting’ after the correct item via a series of approximations, suggesting impaired ‘refreshing’ of the verbal buffer. In addition, patients commonly show tip-of-the-tongue phenomena, consistent with impaired activation of phonological representations [1•, 118, 119] and—to the extent that initial activation of word representations is necessary to access the verbal semantic system [120]—this mechanism might also contribute to the variable semantic deficit in lvPPA [114, 121]. There is some direct fMRI evidence for impaired activation of phonological representations in lvPPA linked to involvement of temporo-parietal junctional cortex [29••], which is both the key locus of auditory-motor phonological transformations in the healthy brain [122], and a primary target of the pathological process in lvPPA [7, 14•, 118]. Although variability of deficits between series may point to separate sub-syndromes within lvPPA [14•], it is possible that this variation is at least in part physiologically based, reflecting the dynamic impact of Alzheimer pathology (the most frequent molecular substrate) on modulatory cholinergic transmission [123].

### nfvPPA

This syndrome is typically considered a predominantly motor disorder of impaired speech and language output and it remains unclear whether there is a ‘pure’ motor sub-syndrome within the nfvPPA spectrum [1•, 4, 9, 10, 114, 124]. However, many if not most patients evolve associated impairments of linguistic processing, whilst the hallmark feature of expressive agrammatism in general signifies a more pervasive disorder of sentence processing [114]. Verbal working memory is also frequently impaired [114]. A candidate, generic unifying mechanism may be impaired temporal scaffolding and/or combinatorial sequencing and scheduling of speech and other sensori-motor routines, particularly where these depend on predictive coding governed by learned ‘rules’. Such a mechanism might underpin the more basic impairments of musical, prosodic, and other auditory pattern analysis recently described in nfvPPA, notably affecting the discrimination of rhythm and regularity [29••, 33, 71, 75••, 76, 125, 126•, 127, 128]. The difficulty that patients with nfvPPA experience in processing speech and other auditory signals may be at least in part attributable to inflexible neural predictions about incoming sensory information and delayed updating of neural templates based on errors [77•]. Such a deficit might account for other, diverse phenomena documented in nfvPPA that on face value are difficult to reconcile with an essentially motoric disorder. These include impaired pure tone perception [74••], degraded speech comprehension [52••], and, in patients’ spontaneous speech, the frequent appearance of ‘binary reversals’ (substitution of closely related words with incorrect polarity, e.g. ‘yes’ for ‘no’) [129]. This pathophysiological formulation accords with neuroanatomical evidence implicating dorsal anterior cingulate and supplementary motor cortex in the impaired analysis of temporal patterns in nfvPPA [29, 76, 127]. More broadly, it fits with the well-documented role played by inferior frontal gyrus, anterior superior temporal gyrus, and anterior insula (the core cortical targets of nfvPPA) in the hierarchical processing of linguistic and non-linguistic sequences [130–135] and the intimate, oscillatory interaction between top-down and bottom-up mechanisms during speech processing [136–138].

## Conclusions and Future Directions

Here, we have outlined recent progress toward a pathophysiology of PPA syndromes. Improved physiological understanding may improve the in vivo prediction of molecular pathologies, improve our ability to monitor treatment response and demonstrate efficacy, and improve the design of targeted symptomatic interventions. These interventions include ‘traditional’ speech and language therapy approaches, but auditory rehabilitation strategies may also hold promise in ameliorating some of the core auditory symptoms outlined above. Despite encouraging recent progress, there are a number of issues that remain to be addressed. First, much work remains to be done in defining new pathophysiological markers of these diseases—progress will depend on an integrated multi-modality approach including functional neuroimaging, neurophysiological, and histopathological techniques, not merely to recapitulate well-established patterns of network disintegration but to identify novel markers of neural reorganisation and plasticity [139]. Second, PPA is rare—if we are to be successful in defining coherent clinico-pathological entities, we will need larger cohorts of patients, which will require large multicentre studies using instruments that are appropriate for speakers of different languages where possible (tests of basic auditory processing may be an attractive prospect here). Third, a validated symptom-led staging system for the major forms of PPA will be vital for tracking disease evolution and planning appropriate care pathways. Finally, the Covid-19 pandemic has changed all of our lives in unprecedented ways, and people with PPA and their caregivers face unique challenges [140••]. The almost universal social dislocations imposed by the virus will ideally motivate clinicians and health care providers to take advantage of the innovative technological ways of working that have been developed: evidence suggests that teletherapy is possible within the context of PPA [141], and perhaps remote- and videoconferencing-based assessments could pave the way for the future national and international collaborations that are needed. Finally, we believe that it may now be time to update the current PPA consensus criteria, taking into account a decade of new research findings and incorporating physiologically informed disease metrics.
